# Comparative transcriptional analysis of hop responses to infection with *Verticillium nonalfalfae*

**DOI:** 10.1007/s00299-017-2177-1

**Published:** 2017-07-11

**Authors:** Vasja Progar, Jernej Jakše, Nataša Štajner, Sebastjan Radišek, Branka Javornik, Sabina Berne

**Affiliations:** 1 Department of Agronomy, Biotechnical Faculty, University of Ljubljana, Ljubljana, Slovenia; 2 Plant Protection Department, Slovenian Institute of Hop Research and Brewing, Žalec, Slovenia

**Keywords:** Hops, Verticillium wilt, Biotic stress, RNA-Seq, Differential gene expression

## Abstract

**Key Message:**

**Dynamic transcriptome profiling revealed excessive, yet ineffective, immune response to** ***V. nonalfalfae*** **infection in susceptible hop, global gene downregulation in shoots of resistant hop and only a few infection-associated genes in roots.**

**Abstract:**

Hop (*Humulus lupulus* L.) production is hampered by Verticillium wilt, a disease predominantly caused by the soil-borne fungus *Verticillium nonalfalfae*. Only a few hop cultivars exhibit resistance towards it and mechanisms of this resistance have not been discovered. In this study, we compared global transcriptional responses in roots and shoots of resistant and susceptible hop plants infected by a lethal strain of *V. nonalfalfae*. Time-series differential gene expression profiles between infected and mock inoculated plants were determined and subjected to network-based analysis of functional enrichment. In the resistant hop cultivar, a remarkably low number of genes were differentially expressed in roots in response to *V. nonalfalfae* infection, while the majority of differentially expressed genes were down-regulated in shoots. The most significantly affected genes were related to cutin biosynthesis, cell wall biogenesis, lateral root development and terpenoid biosynthesis. On the other hand, susceptible hop exhibited a strong defence response in shoots and roots, including increased expression of genes associated with plant responses, such as innate immunity, wounding, jasmonic acid pathway and chitinase activity. Strong induction of defence-associated genes in susceptible hop and a low number of infection-responsive genes in the roots of resistant hop are consistent with previous findings, confirming the pattern of excessive response of the susceptible cultivar, which ultimately fails to protect the plant from *V. nonalfalfae*. This research offers a multifaceted overview of transcriptional responses of susceptible and resistant hop cultivars to *V. nonalfalfae* infection and represents a valuable resource in the study of this plant-pathogen interaction.

**Electronic supplementary material:**

The online version of this article (doi:10.1007/s00299-017-2177-1) contains supplementary material, which is available to authorized users.

## Introduction

Hop (*Humulus lupulus* L.) is a perennial, dioecious plant belonging to the *Cannabaceae* family. It is cultivated commercially for hop products comprising hop cones, powdered or pelleted hops and hop extracts (Haunold 2010). According to the International Hop Growers’ Convention (Paris, France—April 18, 2016), it was grown on 53,876 hectares in 2016, with an estimated worldwide hop production of 105,642 metric tons. Traditionally, hop has been used in the brewing industry as an ingredient that adds characteristic bitterness and aroma to beer, as well as stabilizing the foam and preserving the beer (Steenackers et al. 2015). In recent years, hop-derived compounds have been discovered that exhibit health beneficial biological properties, including anti-oxidative, anti-inflammatory, anti-proliferative and anti-carcinogenic activities, as well as the suppression of osteoporosis and obesity (Van Cleemput et al. 2009; Kirkwood et al. 2013; Li et al. 2015).

Worldwide, hop plantations suffer economic losses due to fungal diseases, such as downy mildew, powdery mildew and Verticillium wilt. The latter disease on hop is predominantly caused by *Verticillium nonalfalfae* (formerly *Verticillium albo*-*atrum*), a soil-borne fungus. The disease symptoms vary depending on the strain’s aggressiveness, from minor wilting symptoms (yellowing and upward curling of leaves, swollen bines and brown discolouration of vascular tissue) caused by the mild pathotype to rapid collapse of leaves and branches, resulting in plant death within a few months after infection with the lethal pathotype (Radišek et al. 2003, 2006). *V. nonalfalfae* released from decaying plants forms melanized hyphae as resting structures, which survive in soil for three to 4 years. On sensing host plant root exudates, these structures germinate into mycelial hyphae, which directly penetrate the root epidermal cells, enter the xylem vessels and spread systemically by the production of spores that are carried through the plant by the transpiration flow (Yadeta and Thomma 2013; Cregeen et al. 2015).

At the early stages of infection by vascular plant pathogens, plant defence relies on protective physical barriers (e.g., Casparian strips, suberin), preformed chemical defence compounds (e.g., glucosinolates, flavonoids, antimicrobial proteins) and general immune responses induced by microbe-associated molecular patterns (MAMPs) (De Coninck et al. 2014). Once the pathogens reach the xylem vessels, they are presumably recognized by specific extracellular receptors in the surrounding parenchyma cells. This recognition activates the formation of tyloses, the accumulation of pectin-rich gels and gums, vascular coating and callose and secondary cell wall depositions (Yadeta and Thomma 2013; De Coninck et al. 2014; Cregeen et al. 2015). A tissue-specific developmental program that leads to the formation of new xylem elements is also observed (Reusche et al. 2012). Furthermore, significant metabolic changes occur and involve the induction of pathogenesis-related (PR) proteins, peroxidases and proteases, as well as the production of phytoalexins, sulphur-containing compounds and phenolic compounds (Yadeta and Thomma 2013; De Coninck et al. 2014). Plant microarray and RNA-Seq studies have revealed that the interaction between vascular wilt pathogens and host plants involves transcriptional reprogramming of hundreds of genes, activation of Ca^2+^-signalling pathways, induction of ROS and MAPK cascades and modulation of phytohormone signalling (Hu et al. 2008; van Esse et al. 2009; Chen et al. 2014).

Transcriptomic studies on compatible and incompatible interactions between *Verticillium* spp. and several hosts have been reported employing data generated by RNA-seq. Comparison of the sea-island cotton (resistant) and upland cotton (susceptible) response to infection with aggressive and mild strains of *V. dahliae* led to the identification of 44 differentially expressed genes (DEGs) with possible implication in the resistance reaction, highlighting genes involved in the phenylpropanoid pathway (Sun et al. 2013), similar to other findings in cotton (Xu et al. 2011), tomato (Gayoso et al. 2010) and wild eggplants (Zhou et al. 2016), in which synthesis of lignin is presented as an important plant defence reaction against infection by *Verticillium* species. Most DEGs found in the compatible interaction of the tomato—*V. dahliae* pathosystem were also associated with phenylpropanoid metabolism (Tan et al. 2015). In the compatible interaction of *Medicago truncatula* and *Verticillium alfalfae*, Toueni et al. (2016) found a disorganized response involving many genes from different functional classes. In contrast, in the incompatible interaction, they identified several genes associated with PTI and hormonal signalling, as well as several transcriptional factors, suggesting that the resistance of the studied *M. truncatula* line is due to innate immunity. Global transcriptome profiling and DEGs analysis were also carried out on sunflower—*V. dahliae* interactions, revealing massive transcriptional reprogramming and classifying resistant cultivar DEGs into plant hormone signalling transduction, plant-pathogen interaction and flavonoid biosynthesis functional categories (Guo et al. 2017).

Although several fungicides have been found to be effective against *Verticillium* spp. in vitro, none have proved successful in field tests (Crowe and Parks 2001). To date, the only measures to control Verticillium wilt are phytosanitary measures, crop rotation and planting resistant crop varieties. In tomato, a leucine-rich repeat receptor-like protein Ve1 was shown to confer resistance against race 1 strains of *V. dahliae* (Fradin et al. 2009), which secrete the Ave1 effector protein (de Jonge et al. 2012). In the following years, several Ve1 homologs were identified within and outside the *Solanaceae* family, indicating an ancient origin of this immune receptor (Song et al. 2016). A functional hop homolog of the immune receptor Ve1 has been reported to mediate resistance against *V. dahliae* but triggers only a weak hypersensitive response when co-expressed with Ave1 in *Nicotiana tabacum* (Song et al. 2016). However, the Ave1 sequence could not be found in hop-infecting strains of *V. nonalfalfae* (unpublished data) and so the Ve1 receptor does not seem to convey resistance to this pathogen in hop.

Understanding the mechanisms of hop resistance to Verticillium wilt and the identification of the participating genes would facilitate marker-assisted selection in hop resistance breeding. Genetic studies of Verticillium wilt resistance in hop have revealed a single genomic QTL region associated with resistance to *V. nonalfalfae*, explaining up to 26% of phenotypic resistance and suggesting the involvement of more than a single gene (Jakše et al. 2013). In a proteomic study of the hop—*V. nonalfalfae* pathosystem, an accumulation of defence-related proteins was detected in the roots of a susceptible cultivar but no significant infection-specific changes were observed in the roots of a resistant cultivar, indicating the involvement of constitutive rather than induced defence mechanisms in resistance to *V. nonalfalfae* (Mandelc et al. 2013). A transcriptional study on the same pathosystem confirmed strong up-regulation of genes for pathogenesis-related proteins in the susceptible cultivar, while genes implicated in ubiquitination and vesicle trafficking were reported to be up-regulated in the resistant and down-regulated in the susceptible cultivar (Cregeen et al. 2015).

In this study, we investigated the dynamics of hop—*V. nonalfalfae* interactions to identify specific defence responses of susceptible and resistant hop cultivars. To demonstrate this, we set up an experiment in which susceptible and resistant hop plants were inoculated with a lethal strain of *V. nonalfalfae* and compared to mock-inoculated samples of shoots and roots at four time points. The samples were subjected to RNA-Seq profiling, differential expression analysis and functional network analysis. The results indicate that the susceptible hop cultivar mounts a strong basal defence response against *V. nonalfalfae*, while in the resistant cultivar there was a remarkably low differential expression in roots and a strong general down-regulation of genes in shoots. In addition to providing a global overview of the response of susceptible and resistant hop plants to *V. nonalfalfae*, the data generated in the course of the study will serve as an invaluable resource for research of Verticillium wilt.

## Materials and methods

### Plant material and colonization

The study was performed on a Verticillium wilt resistant hop cultivar (Wye Target) and a susceptible cultivar (Celeia). One-year-old rooted cuttings of both hop cultivars were inoculated with a lethal PV1 pathotype (T2 isolate) of *V. nonalfalfae* by ten-minute root-dipping in a fungal spore suspension (5 × 10^6^ conidia per ml), while control plants were mock inoculated using sterile water (Flajšman et al. 2017). After root-dipping, the plants were transplanted to a sterile commercial substrate and grown under controlled conditions in a plant growth chamber with a 12 h photoperiod at 22 °C and 65% relative humidity during the light period, and 20 °C and 70% relative humidity during the dark period.

**Fig. 1:**
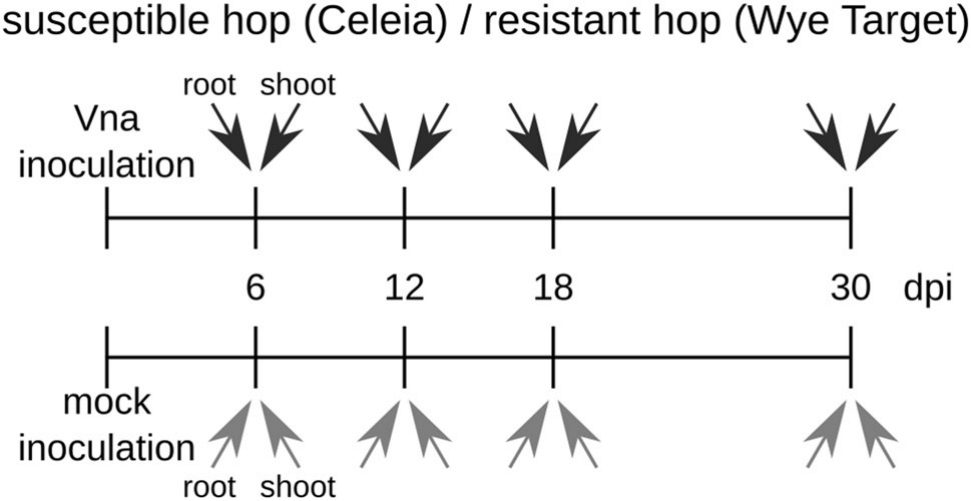
Study design—sampling scheme. The samples were collected from susceptible (Celeia) and resistant (Wye Target) hop cultivars, at 4 time points, for roots and shoots separately. Plants at each condition were either inoculated with *V. nonalfalfae* or mock inoculated, thus obtaining 32 different samples in total

The experimental setup is illustrated in Fig. 1. Root and shoot samples of susceptible and resistant plants were harvested at 6, 12, 18 and 30 days post inoculation. For each condition, both inoculated and mock inoculated plants were collected to be compared for differential expression. Samples from three individual hop plants per condition were ground in liquid nitrogen and stored at −80 °C. To confirm *V. nonalfalfae* infection in the sampled plants, mycological re-isolation of the pathogen was carried out and the presence of fungal DNA was additionally confirmed with qPCR, as described previously (Cregeen et al. 2015).

### RNA isolation

Total RNA was isolated with a Spectrum Plant Total RNA Kit (Sigma-Aldrich) in combination with on-column DNase treatment (On-Column DNase I Digestion Set, Sigma-Aldrich). The concentration and purity were measured on a NanoVue spectrophotometer (GE Healthcare). The quality of isolated RNA was further assessed on the Agilent 2100 Bioanalyzer system (Agilent Technologies) using an Agilent RNA 6000 Nano Kit (Agilent Technologies).

### Sequencing

For sequencing, total RNA from 3 biological replicates per condition was pooled in equal amounts (1 μg per replicate), thus obtaining 32 different samples (4 time points * 2 cultivars * 2 tissues * 2 control/inoculated). mRNA libraries were constructed using an Illumina TruSeq Sample Prep Kit and sequenced on the Illumina Genome Analyzer HiSeq 2000 platform, using a 50 bp single-end read setup at a minimum depth of 20 million reads per sample. The resulting raw nucleotide sequences have been deposited for public availability in the European Nucleotide Archive, collectively under study ID PRJEB14243 (http://www.ebi.ac.uk/ena/data/view/PRJEB14243).

### RNA-Seq mapping and functional annotation

The RNA-Seq pipeline was implemented in CLC Genomics Server 7.0.2 (Qiagen; https://www.qiagenbioinformatics.com/products/clc-genomics-server/) and consisted of trimming low-quality, adaptor-contaminated and under-length sequenced reads and mapping of processed reads (corresponding to a minimum of 90% read length with at least 90% similarity) to the nucleotide sequences of reference draft hop genome *natsume.shinsuwase.v1.0.11262014* (HopBase: A unified genomic database for hop; http://hopbase.cgrb.oregonstate.edu/). Exact trimming and mapping parameters are given in Online Resource 1. The sequence data were also examined against the *Verticillium* genome to ensure that the data were not skewed to expressed genes that share conservation between hop and *Verticillium*.

Functional annotation of gene models from the reference genome was performed with a blastx search (Altschul et al. 1997) against the *Viridiplantae* section of the NCBI *nr* database and by subsequent Blast2GO analysis (Conesa et al. 2005) to assign them Gene Ontology (GO) terms, which were then further mapped to GO Slim Plant terms. Additionally, a blastx search against the *TAIR10 representative* database (TAIR: The *Arabidopsis* Information Resource; https://www.arabidopsis.org/) was performed to associate gene models with TAIR IDs of best matching *Arabidopsis* hits. Gene models were mapped to KEGG *Arabidopsis* pathways based on their associated TAIR IDs.

### Library normalization and determination of differential gene expression

Each of the 32 samples was represented as a library of read counts mapped to 46,735 hop gene models. Read counts were processed using customized scripts in the R software environment (The R Project for Statistical Computing, https://www.r-project.org/). The libraries were first adjusted for composition bias by the trimmed mean of the *M* values normalization (TMM) method (Robinson and Oshlack 2010) using the edgeR package (Robinson et al. 2010) and for size by computing CPM (counts per million—number of reads mapped to a gene model per million reads mapped to the library). Only gene models with CPM >1 in at least one of the samples were considered expressed (23,755 gene models), the remainder were filtered out. To facilitate further analysis, the data were represented as a matrix of log_2_CPM expression values.

Differential gene expression was estimated by computing the area of the region bounded by time series expression profiles of infected and control (mock inoculated) plants, from which the significance of differential expression was determined according to the method described by Di Camillo et al. (2007). The method was applied to all four cultivar-tissue combinations (resistant shoot, resistant root, susceptible shoot and susceptible root). The calculations, ranking of genes and statistics for this method were performed using the R package FunPat (Sanavia et al. 2015). An expression level dependent model was used to estimate the error, and the quantiles of the empirical distribution were chosen for a null hypothesis distribution. The obtained *p* values were adjusted for multiple testing using the default FunPat method. In each cultivar-tissue combination, the genes were considered as differentially expressed genes (DEGs) if *p*_adj._ < 0.05. The results of the FunPat analysis are included in Online Resource 2.

Enrichment of individual cultivar-tissue combinations with GO Slim Plant terms and KEGG pathways was assessed by considering the number of DEGs that were assigned to an individual GO term or KEGG pathway relative to the total number of DEGs in that cultivar-tissue combination.

### Network-based analysis of functional enrichment

We estimated the functional group enrichment of the most differentially expressed genes using a Cytoscape plugin ClueGO (Bindea et al. 2009). For each cultivar-tissue combination, the top 100 DEGs were selected according to their rank assigned by FunPat analysis; for roots of the resistant cultivar, some genes with *p*_adj._ > 0.05 were included to allow comparison with the other combinations. TAIR IDs associated with these genes were used as an input to ClueGO, in which Gene Ontology (GO) terms- and group-enrichment analysis was performed at medium network specificity (GO levels between 3 and 8) using the one-sided hypergeometric test with Bonferroni step-down *p* value correction for multiple testing.

### Identification of significant temporal profiles of differentially expressed genes

To identify significant temporal differential expression profiles of the top 100 DEGs in a time course of each cultivar-tissue combination (the same subset of DEGs as used for network-based enrichment analysis), their log_2_ fold-change (log_2_FC) values for each time point (6, 12, 18 and 30 days post inoculation) were subjected to STEM—Short Time-series Expression Miner software (Ernst and Bar-Joseph 2006). The program was set to assign DEGs to the best fitting temporal profiles, with the following parameters: the maximum number of model profiles was set to 50, the maximum unit change in model profiles between time points to 5 and maximum correlation of model profiles to 0.8. For statistical evaluation, all permutations were used for each DEG and *p* values were adjusted for multiple testing with the Bonferroni correction. Cross references of the DEGs with their associated TAIR IDs were provided to the program to determine the enrichment of significant temporal profiles with GO terms, using TAIR as the gene annotation source. The analysis was performed on GO biological process terms with levels between 3 and 5 and a randomization test with 1000 samples was applied to adjust *p* values for multiple testing.

### Validation of gene differential expression patterns with reverse transcription quantitative PCR

The expression patterns determined by RNA-Seq were validated with reverse transcription quantitative PCR (RT-qPCR) on twelve selected genes. Primers for these genes were designed with Primer3 software (Untergasser et al. 2012) and are listed in Online Resource 1. cDNA synthesis was performed from 1 µg of total RNA in 20 µl reactions using a High-Capacity cDNA Reverse Transcription Kit (Applied Biosystems). The qPCR reactions were carried out on the 7500 Fast Real-Time PCR system (Applied Biosystems) using a SYBR Green PCR Master Mix (Applied Biosystems) and 2 ng cDNA per 10 µl reactions. Three biological and two technical replicates of shoot samples were used for each condition. The relative expression of genes in infected plants compared to mock-inoculated plants was calculated for each cultivar at each time point as _ΔΔ_C_T_, based on the method described by Livak and Schmittgen (2001). Previously validated hop genes YLS8, DRH1 and CAC (Štajner et al. 2013) were used as references.

For comparison between RT-qPCR and RNA-Seq, log_2_FC values for differential expression between infected and control plants were calculated for the selected genes for each method separately and the results were then compared by calculating Pearson’s product moment correlation coefficient (*r*).

## Results

### RNA sequencing and mapping

In this study, we conducted a comprehensive time-course RNA-Seq experiment with infected and mock inoculated plants (Fig. 1) to uncover the global expression changes in shoot and root tissues of resistant and susceptible hop plants following *V. nonalfalfae* colonization.

High-throughput sequencing of 32 hop RNA samples yielded a total of 822.6 M reads of length 50 bp, amounting to over 41 Gbp of raw sequence data. At the trimming step, under 0.5% of reads were removed in each sample, so the final sequencing yield amounted to between 18.3 M and 38.3 M trimmed reads per sample (25.7 M on average). These were then mapped to the reference hop genome from HopBase (http://hopbase.cgrb.oregonstate.edu/) annotated with 46,735 gene models with a mean length of 2347 bp. Cytogenetic research and genome assembly analysis (Hill et al. 2017) revealed that the hop genome is highly repetitive, which causes difficulties in short-read genome assembly and transcript mapping. Based on the lack of a quality reference genome, the mapping rate was expected to be slightly lower. Of the trimmed reads, 49% mapped to exonic regions, 3% to intronic regions and 13% to intergenic regions, while 35% did not map to the reference genome. However, 65% of mapped reads is comparable with other RNA-Seq studies of hop: 70% for ‘Shinsuwase’ (Natsume et al. 2015) and 76% for ‘Teamaker’ (Hill et al. 2017) or other plants infected by Verticillium wilt—50% mapped reads and 80–82% mapped reads in two studies on cotton (Xu et al. 2011; Sun et al. 2013) and 71–89% mapped reads in a study on tomato (Tan et al. 2015). The reads that mapped to exonic regions represented 181.5-fold average coverage of reference gene models. Of 46,735 annotated hop gene models, between 17,562 and 19,430 (37.6 and 41.6%) were expressed (CPM ≥ 1) in individual samples. Of all annotated gene models, 23,755 or 50.8% were expressed in at least one of the 32 conditions and were considered for further analysis to determine their differential expression.

### Differential expression of hop genes following infection with *V. nonalfalfae*

Differential expression of genes in infected plants in comparison to control (mock inoculated) plants was determined for the four cultivar-tissue combinations (resistant shoots, susceptible shoots, resistant roots and susceptible roots) using FunPat, an R package developed for differential expression analysis in time-series experiments (Sanavia et al. 2015). We considered the genes with adjusted *p* value less than 0.05 to be differentially expressed genes (DEGs). The complete list of DEGs for each cultivar-tissue combination, including differential expression data and annotations, is available in Online Resource 2.

**Fig. 2:**
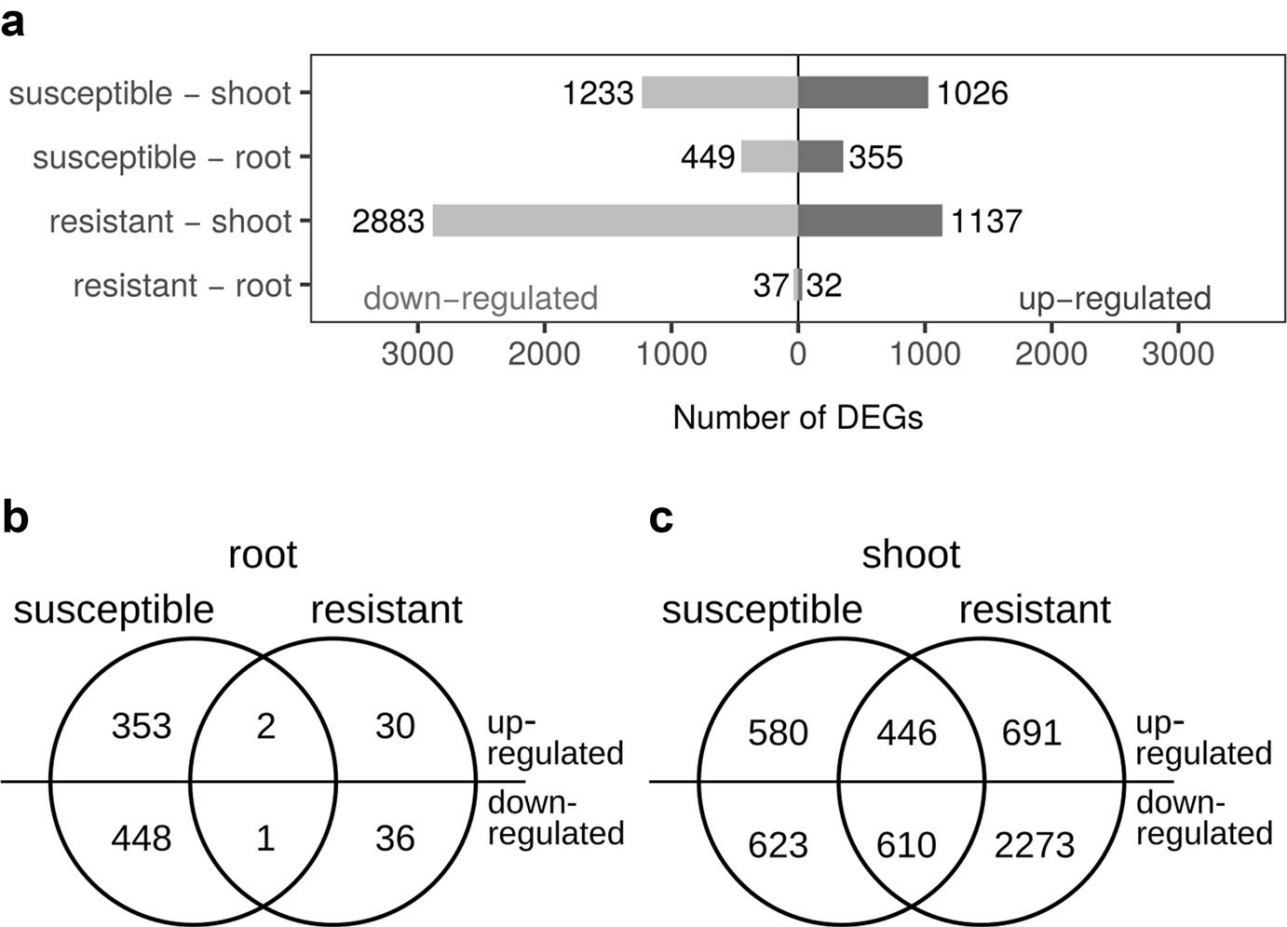
The number of differentially expressed genes (DEGs) in hop plants infected with *V. nonalfalfae* based on pairwise comparison with their respective mock inoculated controls. **a** Numbers of up- and down-regulated DEGs per cultivar-tissue combination. **b** Venn diagram for number of DEGs in roots in common and specific to both cultivars. **c** Venn diagram for number of DEGs in shoots in common and specific to both cultivars. In **b** and **c** the number of up-regulated genes is indicated above the *horizontal line* and the number of down-regulated genes below it

The total number of DEGs varied considerably among the cultivar-tissue combinations (Fig. 2a). There were fewer DEGs in roots than in shoots: in the susceptible cultivar, a total of 804 DEGs was determined in roots and 2259 DEGs in shoots, while in the resistant cultivar there were only 69 DEGs in roots and 4020 DEGs in shoots. In each cultivar-tissue combination, and especially in shoots of the resistant cultivar, more DEGs were down- than up-regulated. In shoots, there were 1056 DEGs common to both cultivars, 446 up-regulated and 610 down-regulated (Fig. 2b), while in roots only two up-regulated and one down-regulated DEGs were common to both cultivars.

### GO Slim and KEGG pathways enrichment of DEGs

**Fig. 3:**
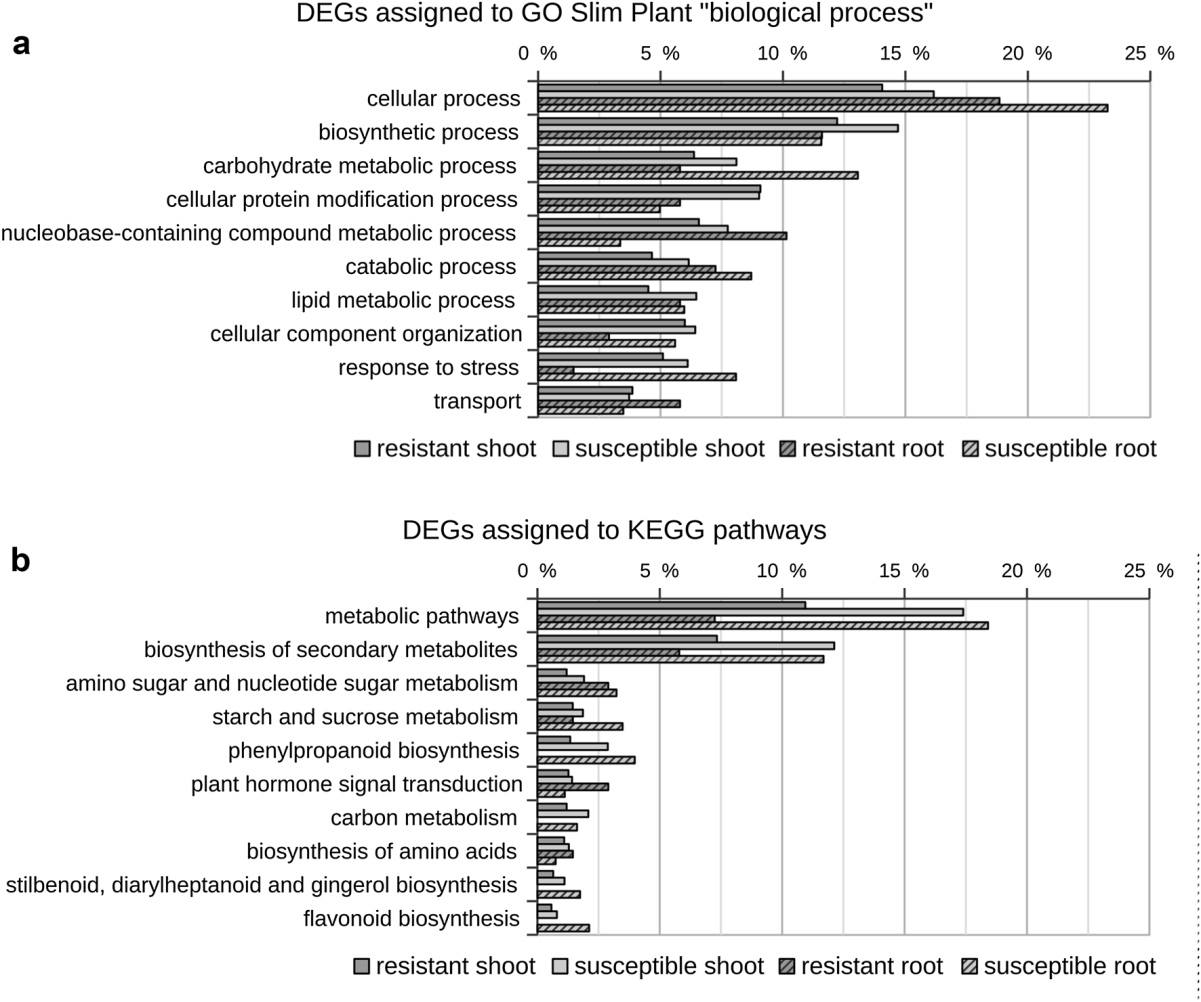
GO Slim Plant term (**a**) and KEGG pathway (**b**) enrichment of differentially expressed hop genes following infection with *V. nonalfalfae*. The *bars* represent percentages of differentially expressed genes (DEGs) assigned to an individual GO term or KEGG pathway relative to the total number of DEGs in a particular cultivar-tissue combination

As a first step towards understanding the functional implications of hop transcriptional response to infection with *V. nonalfalfae*, DEGs were analysed for GO Slim Plant and KEGG enrichment in each cultivar-tissue combination. On average, 73% of DEGs per cultivar-tissue combination were associated with at least one GO Slim term, while 24% were associated with at least one KEGG pathway. The most enriched GO Slim biological process terms and the most enriched KEGG pathways are shown in Fig. 3. The enrichment analysis data for all other GO Slim terms and KEGG pathways are given in tabular format in Online Resource 3.

In the GO Slim category biological process (Fig. 3a), the most enriched terms were ‘cellular process’, to which 14–23% of DEGs were assigned per cultivar-tissue combination, and ‘biosynthetic process’, with 12–15% of DEGs. The term ‘carbohydrate metabolic process’ was enriched with DEGs in roots of the susceptible cultivar (13% of DEGs, compared to 6–8% in the other cultivar-tissue combinations). In roots of the resistant cultivar, 10% of DEGs were assigned to the term ‘nucleobase-containing compound metabolic process’ (compared to 3% in roots of the susceptible cultivar and 7–8% in shoots) and 6% of DEGs to ‘transport’ (compared to 3–4% in other combinations), while only 1% of DEGs were assigned to ‘response to stress’ (compared to 8% in roots of the susceptible cultivar and 5–6% in shoots) and 3% of DEGs to ‘cellular component organization’ (compared to 6% in other combinations).

The most enriched terms in the GO Slim category molecular function (Online Resource 3) were ‘catalytic activity’, to which 10–24% of DEGs were assigned, ‘binding’, which represented 9–20% of DEGs and ‘hydrolase activity’, which represented 9–17% of DEGs. The enrichment of all three terms was greatest in the susceptible cultivar, especially in roots, while they were less enriched in roots of the resistant cultivar. In contrast, the term ‘kinase activity’ was most enriched in roots of the resistant cultivar (9% of DEGs) and less in roots of the susceptible cultivar (3% of DEGs).

In GO Slim category cellular component (Online Resource 3), the most enriched terms were ‘membrane’ with 11–15% of DEGs, ‘plastid’ with 4–14% of DEGs and ‘extracellular region’, to which 3–8% of DEGs were assigned. The terms ‘plastid’ and ‘thylakoid’ were enriched in shoots of the susceptible cultivar (14 and 7% of DEGs, respectively, compared to 4–7 and 1–3% in other cultivar-tissue combinations), while ‘extracellular region’ and ‘cell wall’ were enriched in roots of the susceptible cultivar (8 and 5% of DEGs, respectively, compared to 3–5 and 1–2% in other combinations). In roots of the resistant cultivar, no DEGs were assigned to the terms ‘plasma membrane’ and ‘cytosol’, while in other cultivar-tissue combinations, these terms contained 3–4 and 2–3% of DEGs, respectively.

In KEGG analysis (Fig. 3b), 18 and 17% of DEGs in roots and shoots, respectively, of the susceptible cultivar were mapped to ‘metabolic pathways’ and 7 and 11% of DEGs in roots and shoots of the resistant cultivar. The ‘biosynthesis of secondary metabolism’ pathway was enriched in the susceptible cultivar (12% of DEGs in both roots and shoots compared to 6–7% in the resistant cultivar). Additionally, ‘phenylpropanoid biosynthesis’ was enriched in the susceptible cultivar (3–4% of DEGs were mapped to the pathway, compared to 1% in shoots and no DEGs in roots of the resistant cultivar). Furthermore, in roots of the resistant cultivar, no DEGs were assigned to the pathways ‘carbon metabolism’, ‘stilbenoid, diarylheptanoid and gingerol biosynthesis’ and ‘flavonoid biosynthesis’; however, in other combinations, only 1–2% of DEGs were mapped to these pathways. On the other hand, 3% of DEGs were assigned to the pathway ‘plant hormone signal transduction’ in roots of the resistant cultivars, compared to 1% for other combinations.

### Network-based analysis of functional enrichment

To gain further and more specific insight into the processes in which DEGs are involved, we performed network-based analysis of Gene Ontology enrichment for the top 100 DEGs from each cultivar-tissue combination using Cytoscape plugin ClueGO (Bindea et al. 2009). The results of the analysis for each cultivar-tissue combination are shown in Table 1.

**Table 1:** The results of network-based analysis of functional enrichment for GO terms associated with DEGs in hop after *V. nonalfalfae* infection

GO ID	Susceptible—shoot			GO ID	Resistant—shoot		
	GO Term	**p**[term]	**p**[group]		GO Term	**p**[term]	**p**[group]
GO:0016701	MF—oxidoreductase activity, acting on…	1.7e-4	9.2e-5	GO:0010143	BP—cutin biosynthetic process	7.3e-4	3.6e-4
GO:0080027	BP—response to herbivore	3.5e-6	2.5e-4	GO:0009834	BP—plant-type secondary cell wall biogenesis	7.4e-4	4.1e-3
GO:0031408	BP—oxylipin biosynthetic process	1.8e-5		GO:0042546	BP—cell wall biogenesis	9.0e-3	
GO:0031407	BP—oxylipin metabolic process	1.8e-5		GO:0009832	BP—plant-type cell wall biogenesis	1.4e-2	
GO:0009611	BP—response to wounding	6.6e-4		GO:0016830	MF—carbon–carbon lyase activity	8.5e-3	8.2e-3
GO:0009753	BP—response to jasmonic acid	1.0e-3		GO:0016831	MF—carboxy-lyase activity	2.4e-2	
GO:0006633	BP—fatty acid biosynthetic process	1.6e-2		GO:0016903	MF—oxidoreductase activity, acting on…	2.4e-2	
GO:0008061	MF—chitin binding	1.6e-4	4.0e-4	GO:0043648	BP—dicarboxylic acid metabolic process	2.5e-2	
GO:0004568	MF—chitinase activity	6.2e-4		GO:0010102	BP—lateral root morphogenesis	1.0e-2	1.0e-2
GO:0045087	BP—innate immune response	9.1e-4	4.5e-4	GO:0010101	BP—post-embryonic root morphogenesis	1.0e-2	
GO:0009814	BP—defence response, incompatible interaction	2.4e-3		GO:0048528	BP—post-embryonic root development	1.5e-2	
GO:0009627	BP—systemic acquired resistance	1.3e-2		GO:0048527	BP—lateral root development	2.0e-2	
GO:0016835	MF—carbon–oxygen lyase activity	1.2e-2	1.2e-2	GO:0048645	BP—organ formation	2.2e-2	
GO:0052689	MF—carboxylic ester hydrolase activity	2.1e-2	1.5e-2	GO:0009886	BP—post-embryonic morphogenesis	2.3e-2	
GO:0009642	BP—response to light intensity	2.4e-2	1.6e-2	GO:0031225	CC—anchored component of membrane	2.2e-2	1.1e-2
GO:0010287	CC—plastoglobule	9.4e-4	1.7e-2	GO:0009505	CC—plant-type cell wall	2.4e-2	1.2e-2
GO:0009523	CC—photosystem II	1.4e-2		GO:0000325	CC—plant-type vacuole	2.0e-2	1.3e-2
GO:0019684	BP—photosynthesis, light reaction	1.7e-2					
GO:0009521	CC—photosystem	2.0e-2					

GO ID	Susceptible—root			GO ID	Resistant—root		
	GO Term	**p**[term]	**p**[group]		GO Term	**p**[term]	**p**[group]
GO:0080027	BP—response to herbivore	1.5e-6	7.9e-7	GO:0000272	BP—polysaccharide catabolic process	1.4e-2	9.3e-3
GO:0009611	BP—response to wounding	2.0e-5	1.7e-4	GO:0010333	MF—terpene synthase activity	1.8e-4	8.7e-2
GO:0009753	BP—response to jasmonic acid	3.4e-5		GO:0016838	MF—carbon–oxygen lyase activity, acting on…	2.4e-4	
GO:0031408	BP—oxylipin biosynthetic process	4.0e-4		GO:0016835	MF—carbon–oxygen lyase activity	1.0e-2	
GO:0031407	BP—oxylipin metabolic process	4.0e-4		GO:0000287	MF—magnesium ion binding	1.0e-2	
GO:0051213	MF—dioxygenase activity	1.0e-2		GO:0016114	BP—terpenoid biosynthetic process	1.8e-2	
GO:0008061	MF—chitin binding	7.6e-5	2.2e-4				
GO:0004568	MF—chitinase activity	2.9e-4					
GO:0009814	BP—defence response, incompatible interaction	1.1e-2	6.3e-3				
GO:0048528	BP—post-embryonic root development	1.6e-2	1.1e-2				
GO:0004540	MF—ribonuclease activity	7.8e-3	1.3e-2				
GO:0090305	BP—nucleic acid phosphodiester bond hydrolysis	9.2e-3					
GO:0090501	BP—RNA phosphodiester bond hydrolysis	1.0e-2					
GO:0004518	MF—nuclease activity	1.6e-2					
GO:0000325	CC—plant-type vacuole	1.9e-2	1.4e-2				

In the susceptible cultivar, the enrichment of GO terms with DEGs was similar between shoots and roots. The majority of enriched GO terms were associated with defence response and included terms such as ‘response to wounding’, ‘response to jasmonic acid’, ‘defence response, incompatible interaction’ and ‘chitinase activity’ in both roots and shoots and also ‘innate immune response’ and ‘systemic acquired resistance’ in shoots. Interestingly, none of these terms were found to be enriched in the resistant cultivar. Unique to the roots of the susceptible cultivar was a group of GO terms associated with ribonuclease activity, while enrichment of a group containing photosynthesis-related GO terms was specific to the shoots of the susceptible cultivar (Table 1).

Functional enrichment in the resistant cultivar was different between roots and shoots and to that in the susceptible cultivar. In roots, the GO term ‘polysaccharide catabolic process’ and a group associated with terpenoid biosynthesis were enriched with DEGs, while the most significantly enriched GO terms in shoots were ‘cutin biosynthetic process’ and a group containing GO terms associated with (secondary) cell wall biogenesis, followed by a group involved in dicarboxylic acid metabolism, a group related to lateral root development and GO cellular compartment terms associated with membrane, cell wall and vacuole (Table 1).

### Significant temporal profiles of differentially expressed genes

STEM analysis (Ernst and Bar-Joseph 2006) was used to identify coordinate differential expression of hop genes during the time course of *V. nonalfalfae* colonization. Among the top 100 DEGs analysed for each cultivar-tissue combination, nine significant temporal profiles (*p*_adj._ < 0.05) were identified: 2 and 4 profiles in shoots and roots, respectively, of the susceptible cultivar and 3 in shoots of the resistant cultivar (Online Resource 5), while there were no significant time-course profiles in roots of the resistant cultivar. The DEGs belonging to each of the significant temporal profiles and the enrichment of individual profiles with GO terms are listed in Online Resource 6.

**Fig. 4:**
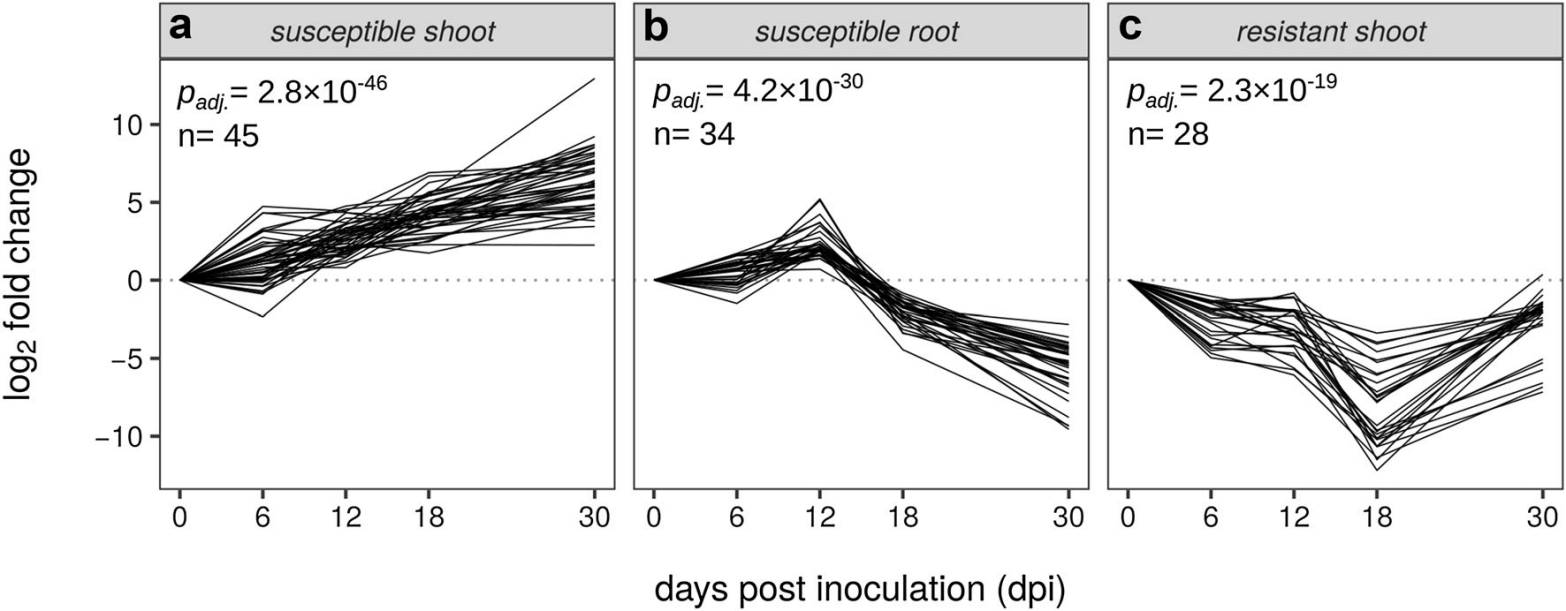
The most significant temporal differential expression profiles of the top 100 DEGs in shoots of the susceptible hop (**a**), in roots of the susceptible hop (**b**) and in shoots of the resistant hop (**c**) after *V. nonalfalfae* infection. For each profile, the number of assigned DEGs out of the top 100 for the corresponding cultivar-tissue combination and their adjusted *p* values are given

Figure 4 shows the most significant temporal profiles of DEGs in shoots and roots of the susceptible cultivar and in shoots of the resistant cultivar. The profile in shoots of the susceptible cultivar, to which 45 out of 100 DEGs in that cultivar-tissue combination were mapped, was characterized by a constantly increasing up-regulation during the course of the experiment (Fig. 4a). Although there were no significantly enriched GO terms for this profile in this cultivar-tissue combination, some GO terms were associated with more than half of the DEGs in this temporal profile, such as ‘response to stress’ and ‘response to biotic stimulus’ (Online Resource 6). The same temporal profile was significant in roots of the susceptible cultivar, in which 21 of 100 DEGs were mapped to it (*p*_adj._ = 1.7 × 10^−14^; Online Resource 5). In this cultivar-tissue combination, 9 GO terms were significantly enriched (*p*_adj._ < 0.05) and included ‘innate immune response’ and ‘defence response to fungus, incompatible interaction’ (Online Resource 6).

However, the most significant temporal profile in roots of the susceptible cultivar, to which 34 of 100 DEGs were mapped, encompassed both up- and down-regulation: starting with little differential regulation at 6 dpi, it exhibited up-regulation at 12 dpi and then increasing down-regulation at 18 and 30 dpi (Fig. 4b). Two GO terms were significantly enriched for this profile: ‘lipid metabolic process’ with 11 associated DEGs and ‘small molecule metabolic process’ with 10 DEGs (Online Resource 6). This temporal profile was also significant in shoots of the susceptible cultivar (*p*_adj._ = 1.6 × 10^−8^) and included 15 out of 100 DEGs (Online Resource 5). The assortment of GO terms associated with this profile was similar to that in roots (Online Resource 6), but none were significantly enriched.

The most significant temporal profile in shoots of the resistant cultivar, which included 28 out of 100 DEGs, showed consistent down-regulation, which was especially pronounced at 18 dpi (Fig. 4c). It was not significantly enriched with any GO term but it included, for example, 9 DEGs associated with ‘lipid metabolic process’, 6 associated with ‘defence response’ and 5 associated with ‘cell wall organization or biogenesis’ (Online Resource 6).

There were four more significant temporal profiles: two in roots of the susceptible cultivar, with 10 and 8 out of 100 DEGs and with a general upward tendency, while a further two were in shoots of the resistant cultivar, with 15 and 10 out of 100 DEGs and with a general downward tendency (Online Resource 5). None of them were significantly enriched with any GO terms (Online Resource 6).

### Validation of RNA-Seq expression patterns with quantitative reverse transcription polymerase chain reaction

**Fig. 5:**
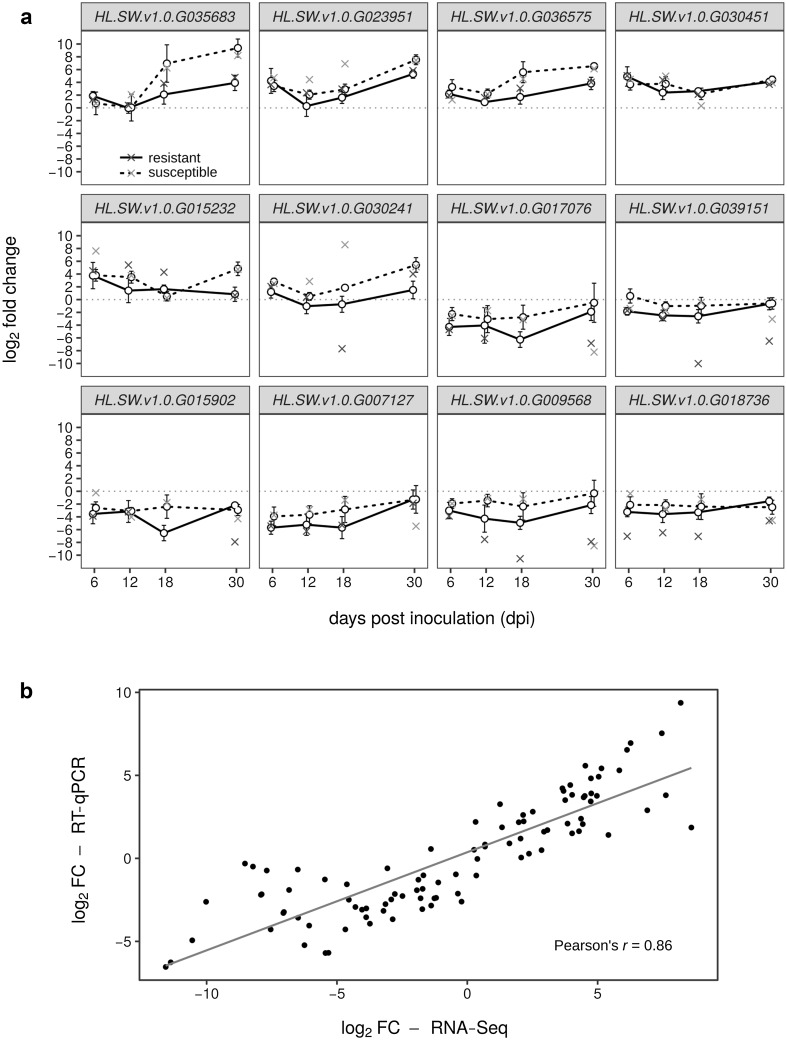
Reverse transcription quantitative polymerase chain reaction (RT-qPCR) analysis of selected hop genes differentially expressed during *V. nonalfalfae* infection and comparison with RNA-Seq results. **a** Plots represent log_2_ fold changes between expression in infected and mock-inoculated plants determined with RT-qPCR and using previously validated reference genes Yellow leaf specific protein 8, *YLS8*, DEAD box RNA helicase, *DRH1*, and Clathrin adaptor complexes medium subunit, *CAC* (Štajner et al. 2013). *Error-bars* indicate standard error (*n* = 3). The *crosses* represent log_2_FC values as determined by RNA-Seq. **b** Comparison of log_2_FC determined by RNA-Seq and RT-qPCR using Pearson’s product moment correlation coefficient. The *points* represent individual measurements of the same sample by the two methods and a trend line is added

To validate the reliability of expression patterns obtained by RNA-Seq, we performed quantitative reverse transcription polymerase chain reaction (RT-qPCR) on twelve selected DEGs in shoots of both cultivars using three biological replicates for each condition. The genes were selected from among those that were determined by FunPat as differentially expressed in at least one of the cultivar-tissue combinations and so that half of them were predominantly up- and half predominantly down-regulated (Table 2). The results of RT-qPCR analysis for the selected genes are shown in Fig. 5a and are also available in tabular format in Online Resource 4.

**Table 2:** Candidate hop genes expressed in *V. nonalfalfae*-interactions and their best *Arabidopsis* blastx matches

HopBase ID	Expression trend	Best TAIR hit	
*HL.SW.v1.0.G035683*	Up	*AT4G33720*	CAP superfamily protein
*HL.SW.v1.0.G023951*	Up	*AT5G43580*	Unusual serine protease inhibitor
*HL.SW.v1.0.G036575*	Up	*AT2G38540*	Lipid transfer protein 1
*HL.SW.v1.0.G030451*	Up	*AT5G24080*	Protein kinase superfamily protein
*HL.SW.v1.0.G015232*	Up	*AT3G50980*	Dehydrin xero 1
*HL.SW.v1.0.G030241*	Up	*AT2G45220*	Pectin methylesterase 17
*HL.SW.v1.0.G017076*	Down	*AT1G24020*	MLP-like protein 423
*HL.SW.v1.0.G039151*	Down	*AT5G15310*	MYB domain protein 16
*HL.SW.v1.0.G015902*	Down	*AT5G45950*	GDSL esterase/acyltransferase/lipase
*HL.SW.v1.0.G007127*	Down	*AT5G33370*	GDSL-like lipase/acylhydrolase superfamily protein
*HL.SW.v1.0.G009568*	Down	*AT2G45970*	CYP86A8, lacerata
*HL.SW.v1.0.G018736*	Down	*AT1G49430*	Long-chain acyl-CoA synthetase 2

To evaluate the correlation between the two methods statistically, we calculated Pearson’s product moment correlation coefficient (*r*) between the log_2_FC values obtained by RNA-Seq and the average log_2_FCs obtained by RT-qPCR (Fig. 5b). The correlation coefficient was 0.86 with a *p* value <2.2 × 10^−16^ and 95% confidence interval between 0.80 and 0.90, indicating a significant and strong correlation between the methods, thus validating the results obtained by RNA-Seq on which the functional enrichment analyses were performed.

## Discussion

This study is the first large-scale transcriptomic RNA-Seq analysis of hop response to infection with *Verticillium nonalfalfae*, the main causative agent of Verticillium wilt in hops. In an extensive time-course survey, the transcriptional responses in roots and shoots of both resistant and susceptible cultivars were determined in infected and healthy plants. The expression levels were monitored at 6, 12, 18 and 30 dpi to match the colonization dynamics of *V. nonalfalfae* in hop, from initial colonization to full symptom development in the susceptible cultivar between 20 and 30 dpi (Cregeen et al. 2015; Flajšman et al. 2017). After root penetration and initial spread of the fungus in the roots of the resistant cultivar, a decline in fungal biomass is observed around 20 dpi due to plant resistant responses and further growth in hop stem is very restricted. Similar systemic colonization in susceptible plants, and restriction of the pathogen to the roots and stem base of resistant plants, has also been reported for *V. longisporum* in oilseed rape (Eynck et al. 2009) and for *V. dahliae* in olives (Markakis et al. 2010).

In the present study, a large amount of expression data was generated during the experiment and processed with a state of the art bioinformatics pipeline to gain insight into functional enrichment of biological processes with differentially expressed genes. The most prominent difference between the cultivars was that the susceptible plants exhibited a much stronger general defence response than the resistant ones, and the cultivars also exhibited differences in response of genes involved in the jasmonate pathway and regulation of photosynthesis, as well as terpenoid biosynthesis and cell wall metabolism.

Differentially expressed genes (DEGs) were determined with FunPat (Sanavia et al. 2015) for four separate cultivar-tissue combinations (shoots and roots of the resistant and the susceptible hop) by comparing the temporal profiles of gene expression in infected plants with those in the control (mock inoculated). Validation of the expression patterns of DEGs with RT-qPCR on twelve selected DEGs resulted in a significant correlation between the methods, with Pearson’s correlation coefficient of *r* = 0.86, which is comparable to transcriptomic study of cotton—*V. dahliae* interaction (Xu et al. 2011). In the roots of the resistant cultivar, there were far fewer DEGs than in the roots of the susceptible cultivar. This is consistent with the results of a proteomic study on the same pathosystem (Mandelc et al. 2013), in which 252 infection-specific protein spots were discovered in the susceptible hop and no changes in the resistant cultivar after infection with *V. nonalfalfae*, possibly indicating the involvement of constitutive rather than induced defence mechanisms in hop resistance to the fungus. A less intense response in roots of the resistant compared to the susceptible cultivar is also reported in transcriptomic studies of Verticillium wilt on other hosts. In a study of tomato infected by *Verticillium dahliae* (van Esse et al. 2009), the microarray results indicated a similar disproportion between the number of DEGs in roots of resistant and susceptible plants at 3 and 7 dpi: there were 147 DEGs in roots of the resistant compared to 1188 DEGs in roots of the susceptible tomato plants. Similarly, in *Medicago truncatula* infected by *Verticillium alfalfae* (Toueni et al. 2016), the response in roots of a susceptible cultivar was stronger, with 4053 detected DEGs, compared to 1055 DEGs detected in roots of a resistant cultivar. These findings suggest that susceptibility to *Verticillium* infection is not due to lack of immune responses, but is rather caused by some failure in the efficacy of the responses.

Enrichment analysis of plant biologic and metabolic processes with GO Slim and KEGG pathways revealed a higher percentage of DEGs in the roots of the susceptible hop associated with ‘carbohydrate metabolic process’ and ‘hydrolase activity’, ‘biosynthesis of secondary metabolites’, ‘phenylpropanoid biosynthesis’ and ‘response to stress’. On the other hand, in the roots of the resistant hop, a higher percentage of DEGs were attributed to ‘nucleobase-containing compound metabolic process’, ‘transport’, ‘kinase activity’ and ‘plant hormone signal transduction’. In contrast to our results, previous transcriptome studies of the *V. dahliae* interaction with cotton (Xu et al. 2011) and with tomato (Gayoso et al. 2010) detected up-regulation of phenylpropanoid metabolism and synthesis of lignins as prominent processes contributing to a successful defence response of resistant plants. The differences could be attributed to a different mechanism of resistance in those pathosystems, especially since resistance in tomato is mediated by Ve1 receptor-like protein (Fradin et al. 2009; Gayoso et al. 2010).

A more profound insight into the functional implications of hop response to *V. nonalfalfae* was provided using STEM (Ernst and Bar-Joseph 2006) and network-based analysis of functional enrichment by ClueGo (Bindea et al. 2009). Interestingly, a significant temporal profile, with increasing gene expression of PR-4 homologs and thaumatin, was detected only in the susceptible hop and corroborated by network-based enrichment of GO terms such as ‘innate immune response’, ‘defence response, incompatible interaction’, ‘response to stress’ and ‘chitinase activity’. These results are consistent with previous studies of the hop—*V. nonalfalfae* interaction on both proteomic (Mandelc et al. 2013) and transcriptomic levels (Cregeen et al. 2015), showing an accumulation of defence-related proteins (such as chitinases, β-glucanases and thaumatin-like proteins) and steadily increasing expression of genes for pathogenesis-related proteins only in the susceptible hop. Intensive up-regulation of defence-related genes in susceptible but not in resistant plants was also observed in tomato following infection with *V. dahliae* (Robb et al. 2012). Taken together, these findings indicate that a substantial general defence response is mounted in the susceptible plants attacked by *Verticillium* and it intensifies during the time-course of colonization but is ultimately ineffective in protecting the plant from the fungus.

We identified another group of significantly enriched processes in both shoots and roots of the susceptible cultivar, which included GO terms such as ‘response to jasmonic acid’ and ‘oxylipin metabolic process’, and found a corresponding significant temporal profile with peak of gene expression at 12 dpi. DEGs in this profile were, for example, homologs of lipoxygenase 2 and allene oxide synthase, which are also important members of the jasmonic acid biosynthesis pathway (Wasternack and Song 2016). These results suggest a potential involvement of jasmonic acid signalling in the hop interaction with *V. nonalfalfae*. In addition, it has been shown that *V. longisporum* interferes with the jasmonic acid signalling pathway in *Arabidopsis thaliana* by direct interaction with jasmonic acid receptor COI1, thus enhancing the susceptibility of the host (Ralhan et al. 2012). Similar jasmonic acid signalling interference was reported in the interaction between *A. thaliana* and *Fusarium oxysporum* (Thatcher et al. 2009).

GO term enrichment of processes related to photosynthesis was also of interest. Consistent with global down-regulation of photosynthesis in lettuce in response to *V. dahliae* colonization (Klosterman et al. 2011) and other studies of plant-pathogen interactions (Bilgin et al. 2010), several DEGs associated with photosynthesis (e.g., chlorophyll A/B binding protein 1 and photosystem I light harvesting complex gene 2) were down-regulated in shoots of the susceptible hop.

In roots of the resistant cultivar, we found no significant temporal expression profiles and detected only two enriched processes, polysaccharide catabolic process and terpenoid biosynthesis. In addition to antibacterial and antifungal properties of root-secreted terpenoid compounds (Baetz and Martinoia 2014), a strong production of terpenoids, especially in roots of a resistant cultivar, has also been reported in cotton plants infected with *V. dahliae* (Daayf et al. 1997) and in roots of tomato seedlings infected with *V. albo*-*atrum* (Hutson and Smith 1983). On the other hand, certain monoterpenes can stimulate germination of *V. longisporum*, and thus actually enhance infection of *Arabidopsis* roots (Roos et al. 2015).

In shoots of the resistant cultivar, according to network-based functional analysis, various processes were enriched, including modification of cell wall and biosynthesis of cutin. A significant temporal profile, with the strongest gene down-regulation at 18 dpi, was linked to these processes and among others comprised gene homologs of cell wall associated pectinesterases and fasciclin-like proteins, as well as homologs of cutin metabolism associated GDSL lipases and long-chain acyl-CoA synthetase. In addition to serving as a preformed physical barrier, the cell wall responds dynamically to biotic stress by the formation of lignin and suberin, depositions of callose and cross-linking and remodelling of the cell wall structure (Bellincampi et al. 2014). It undergoes significant modifications also due to pathogen released cell wall-degrading enzymes, which have been clearly associated with pathogenicity of *V. dahliae* (Gharbi et al. 2015). Cutin metabolism was the most significantly enriched GO process in resistant hop. Cutin is polyesters of mostly oxygenated fatty acids and a major component of the plant cuticle (Fich et al. 2016). Although the disruption of cutin metabolism in *A. thaliana* increased cuticle permeability and led to strong resistance against necrotrophic fungus *Botrytis cinerea* (Bessire et al. 2007), we did not expect a correlation between the vascular pathogen *V. nonalfalfae* and resistance response related to the cuticle.

In conclusion, this study is the first transcriptome-wide RNA-Seq study of resistant and susceptible hop response to infection with *V. nonalfalfae*, which causes a devastating disease hampering the production of hop. The results suggest a strong general defence response in both shoots and roots of the susceptible cultivar, which is in accordance with the findings of previous studies on the same pathosystem, as well as of studies of Verticillium wilt on some other hosts. A vast amount of expression data was generated and processed in this study, providing a global and multifaceted overview of hop response to *V. nonalfalfae*, encompassing both susceptible and resistant plant responses in shoots and roots during the course of colonization. It is thus a useful addition to research of hop resistance to *V. nonalfalfae* and will contribute to eventual identification of its underlying mechanisms, with potential use in hop breeding programmes in the future.

### **Author contribution statement**

BJ conceived and coordinated the study and BJ, SB, NŠ and JJ participated in its design. SR prepared the plant and fungal material and performed hop inoculations. VP harvested and processed the samples. VP performed RT-qPCR analyses together with NŠ and bioinformatics analysis together with JJ. SB, VP and BJ analysed and interpreted the data and wrote the manuscript. All authors read and approved the manuscript.

## Electronic supplementary material

Below is the link to the electronic supplementary material.

Online Resource 1: Parameters used in processing sequenced reads and primers for RT-qPCR. This PDF file contains detailed parameters that were used for read trimming and mapping in CLC, as well as sequences of primers used for RT-qPCR. Supplementary material 1 (DOCX 17 kb)

Online Resource 2: List of DEGs for each cultivar-tissue combination. This file is an XLSX spreadsheet containing the DEGs (*p*_adj._ < 0.05) for each cultivar-tissue combination in separate sheets. For roots of the resistant cultivar, the top 100 DEGs are given, although some have *p*_adj._ > 0.05 (those are greyed out). The report tables contain HopBase IDs, TAIR IDs and descriptions of the best TAIR10 blastx hits, Entrez IDs and a description of the best NCBI *nr* blastx hits, assigned GO and GO Slim terms and KEGG pathways, DEG ranks and *p*_adj._ values determined by FunPat and log_2_FC values for each time point (colour-coded in heat-plot fashion for easier review). Supplementary material 2 (XLSX 1410 kb)

Online Resource 3: GO Slim and KEGG pathway enrichment of DEGs. This file is an XLSX spreadsheet containing the number and relative percentage of DEGs assigned in each cultivar-tissue combination to GO Slim Plant terms (biological process, molecular function, cellular component) and KEGG pathways, in separate sheets. The percentages are colour-coded for easier review. Supplementary material 3 (XLSX 35 kb)

Online Resource 4: RT-qPCR results for selected genes. This file is an XLSX spreadsheet containing log_2_FC values (mean and standard error for three biological replicates) of expression of selected genes in the shoots of both cultivars following infection with *V. nonalfalfae,* compared to mock inoculated plants of the respective cultivar. Supplementary material 4 (XLSX 12 kb)

Online Resource 5: All significant temporal differential expression profiles of the top 100 DEGs in individual cultivar-tissue combinations. This PNG file includes temporal profiles that were found significant: two in shoots and four in roots of the susceptible cultivar, and three in shoots of the resistant cultivar. The numbers of assigned DEGs out of the top 100 for corresponding cultivar-tissue combination are given, along with their adjusted *p* values. Supplementary material 5 (PNG 703 kb)

Online Resource 6: The genes and GO biological process terms associated with significant temporal differential expression profiles. This XLSX spreadsheet includes multiple sheets – the first contains lists of genes for each significant temporal profile and the following sheets contain GO enrichment data with adjusted *p* values for each profile. Supplementary material 6 (XLSX 30 kb)
