# Supplementary material for: Comparative transcriptional analysis of hop responses to infection with Verticillium nonalfalfae
Source: Plant Cell Rep. 2017 Jul 11;36(10):1599–613. doi: 10.1007/s00299-017-2177-1 (PMC5602066; doi:10.1007/s00299-017-2177-1)
Supplement: Supplementary file 1 — Online Resource 1: Parameters used in processing sequenced reads and primers for RT-qPCR. This PDF file contains detailed parameters that were used for read trimming and mapping in CLC, as well as sequences of primers used for RT-qPCR. Supplementary material 1 (DOCX 17 kb) [file 299_2017_2177_MOESM1_ESM.docx]

## RNA-Seq read trimming and mapping parameters

**Supplementary Table 1** Parameters used for trimming sequenced reads in CLC

| **Ambiguous trim** | Yes |
| --- | --- |
| **Ambiguous limit** | 2 |
| **Quality trim** | Yes |
| **Quality limit** | 0.05 |
| **Use colorspace** | No |
| **Also search on reversed sequence** | Yes |
| **Save discarded sequences** | Yes |
| **Remove 5' terminal nucleotides** | No |
| **Minimum number of nucleotides in reads** | 20 |
| **Discard short reads** | Yes |
| **Remove 3' terminal nucleotides** | No |
| **Trim adapter list** | TruSeq-adapters |
| **Discard long reads** | No |

**Supplementary Table 2** Parameters used in mapping sequenced reads to hop draft genome in CLC

| **Reference type** | Genome annotated with genes and transcripts |
| --- | --- |
| **Reference sequence** | natsume.shinsuwase.v1.0.20141126 |
| **Gene track** | natsume.shinsuwase.v1.0.20141126 (Gene) |
| **mRNA track** | natsume.shinsuwase.v1.0.20141126 (mRNA) |
| **Mapping type** | Also map to inter-genic regions |
| **Mismatch cost** | 2 |
| **Insertion cost** | 3 |
| **Deletion cost** | 3 |
| **Length fraction** | 0.9 |
| **Similarity fraction** | 0.9 |
| **Global alignment** | No |
| **Strand specific** | Both |
| **Maximum number of hits for a read** | 10 |
| **Count paired reads as two** | No |

## RT-qPCR primers

**Supplementary Table 3** Primer sequences used for RT-qPCR on selected genes

| **HopBase ID** | **Primer 1** | **Primer 2** | **Efficiency (%)** |
| --- | --- | --- | --- |
| HL.SW.v1.0.G035683 | GGGTGAATGAGAAGGCCAAC | TTAGCACACCCCAAACGAAC | 99.4 |
| HL.SW.v1.0.G023951 | GGAGAAGAAGCAAAGGAAACGA | CCCAAACCCTAACCCTCTCA | 101.6 % |
| HL.SW.v1.0.G036575 | GACAGCTGTGGTTGCATTGA | CGGTGTCAGCCAGTTTGTC | 97.0 % |
| HL.SW.v1.0.G030451 | CCATGTCAGAATCCGGCAAC | TCGGGAGCAAAGTGTCTGAT | 98.9 % |
| HL.SW.v1.0.G015232 | TGAGGATGATGGGCAAGGAG | TCCATAACTCGGAGCACTGC | 99.7 % |
| HL.SW.v1.0.G030241 | ACCCACCCAACAAAACCAAC | GACGATTTCAGGTCCGAAGC | 98.9 % |
| HL.SW.v1.0.G017076 | CAAGTCCAGCGTTCAAGTGG | ATCCTTGACGACTGTTGGGT | 102.6 % |
| HL.SW.v1.0.G004467 | AAGTTCAGAGGCGTCAGACA | TCCTCGGCTGTCTCAAATGT | 100.1 % |
| HL.SW.v1.0.G039151 | ATATCATCGGCTCGGCTTCA | AGTACGGGCATTGCAACATC | NA |
| HL.SW.v1.0.G007127 | ACTTTGAGCAGTTTTGGCCA | TGGCTGTGGTGGCTAGATAG | 97.3 % |
| HL.SW.v1.0.G009568 | TCCTTGAAACGGCTCAGCTA | AAAGCGTTTGTGGGTCCTTC | 98.1 % |
| HL.SW.v1.0.G018736 | AGATGGCTTGCTTGAGTTGC | CTTTAATCTGGCGTCGACCC | 98.8 % |
