# Supplementary figures and images for: Comparative transcriptional analysis of hop responses to infection with Verticillium nonalfalfae
Source: Plant Cell Rep. 2017 Jul 11;36(10):1599–613. doi: 10.1007/s00299-017-2177-1 (PMC5602066; doi:10.1007/s00299-017-2177-1)

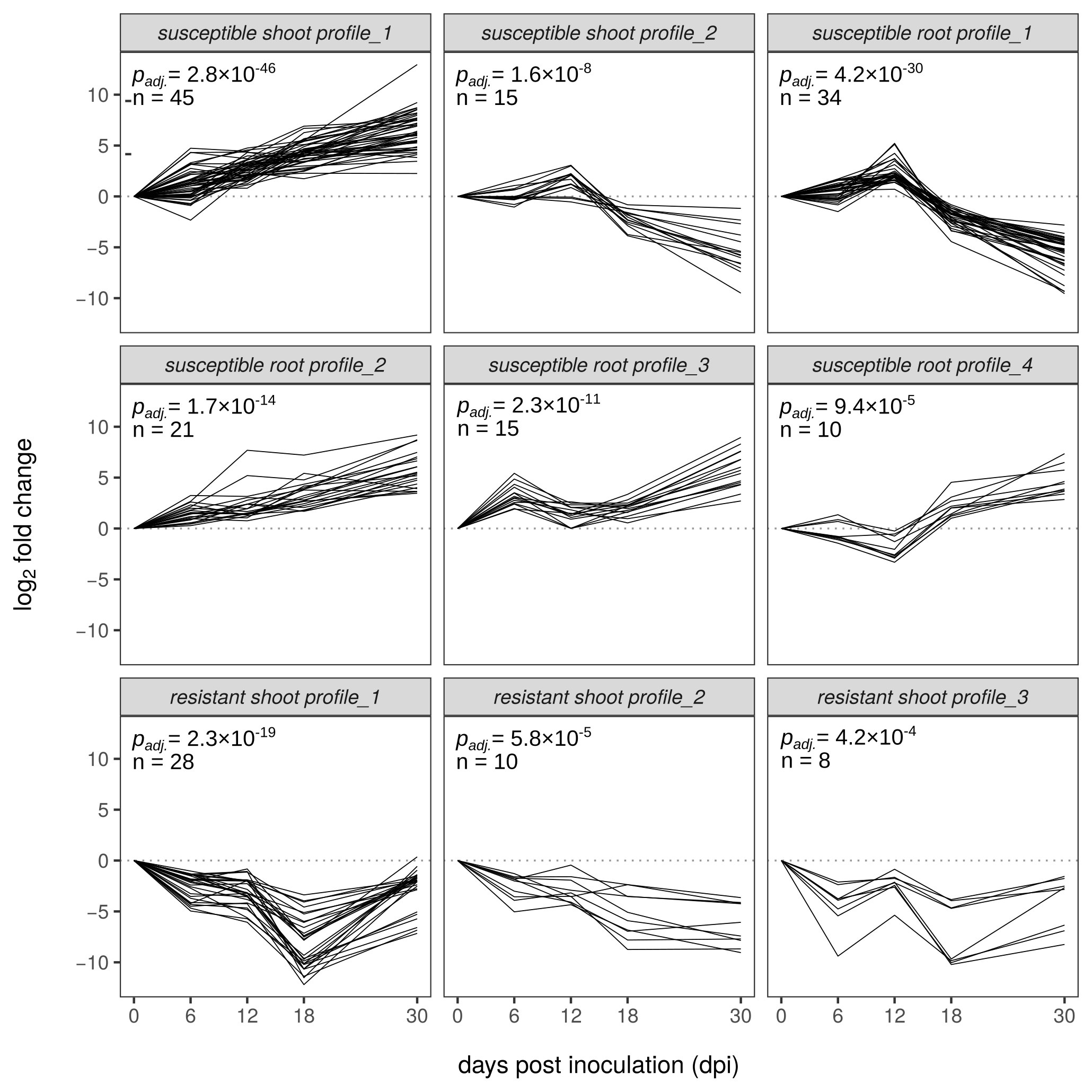

Supplement: Supplementary file 5 — Online Resource 5: All significant temporal differential expression profiles of the top 100 DEGs in individual cultivar-tissue combinations. This PNG file includes temporal profiles that were found significant: two in shoots and four in roots of the susceptible cultivar, and three in shoots of the resistant cultivar. The numbers of assigned DEGs out of the top 100 for corresponding cultivar-tissue combination are given, along with their adjusted p values. Supplementary material 5 (PNG 703 kb) [file 299_2017_2177_MOESM5_ESM.png]
